# Genomic surveillance of *Neisseria gonorrhoeae* to investigate the distribution and evolution of antimicrobial-resistance determinants and lineages

**DOI:** 10.1099/mgen.0.000205

**Published:** 2018-07-31

**Authors:** Koji Yahara, Shu-ichi Nakayama, Ken Shimuta, Ken-ichi Lee, Masatomo Morita, Takuya Kawahata, Toshiro Kuroki, Yuko Watanabe, Hitomi Ohya, Mitsuru Yasuda, Takashi Deguchi, Xavier Didelot, Makoto Ohnishi

**Affiliations:** ^1^​ Antimicrobial Resistance Research Center, National Institute of Infectious Diseases, Tokyo, Japan; ^2^​ Department of Bacteriology I, National Institute of Infectious Diseases, Tokyo, Japan; ^3^​ Virology Section, Division of Microbiology, Osaka Institute of Public Health, Osaka, Japan; ^4^​ Department of Microbiology, Kanagawa Prefectural Institute of Public Health, Kanagawa, Japan; ^5^​ Department of Urology, Graduate School of Medicine, Gifu University, Gifu, Japan; ^6^​ Department of Infectious Disease Epidemiology, Imperial College, London, UK; ^†^​ Present address: Faculty of Veterinary Medicine, Okayama University of Science, 1-3, Ikoinooka, Imabari, Ehime 794-8555, Japan.

**Keywords:** antimicrobial resistance, genomic epidemiology, phylogeny, recombination, *Neisseria gonorrhoeae*, surveillance, cephalosporin, macrolide, fluoroquinolone, coalescent

## Abstract

The first extensively drug resistant (XDR) *Neisseria gonorrhoeae* strain with high resistance to the extended-spectrum cephalosporin ceftriaxone was identified in 2009 in Japan, but no other strain with this antimicrobial-resistance profile has been reported since. However, surveillance to date has been based on phenotypic methods and sequence typing, not genome sequencing. Therefore, little is known about the local population structure at the genomic level, and how resistance determinants and lineages are distributed and evolve. We analysed the whole-genome sequence data and the antimicrobial-susceptibility testing results of 204 strains sampled in a region where the first XDR ceftriaxone-resistant *N. gonorrhoeae* was isolated, complemented with 67 additional genomes from other time frames and locations within Japan. Strains resistant to ceftriaxone were not found, but we discovered a sequence type (ST)7363 sub-lineage susceptible to ceftriaxone and cefixime in which the mosaic *penA* allele responsible for reduced susceptibility had reverted to a susceptible allele by recombination. Approximately 85 % of isolates showed resistance to fluoroquinolones (ciprofloxacin) explained by linked amino acid substitutions at positions 91 and 95 of GyrA with 99 % sensitivity and 100 % specificity. Approximately 10 % showed resistance to macrolides (azithromycin), for which genetic determinants are less clear. Furthermore, we revealed different evolutionary paths of the two major lineages: single acquisition of *penA* X in the ST7363-associated lineage, followed by multiple independent acquisitions of the *penA* X and XXXIV in the ST1901-associated lineage. Our study provides a detailed picture of the distribution of resistance determinants and disentangles the evolution of the two major lineages spreading worldwide.

## Data Summary

1. Genome read data for all samples has been deposited into the DNA Data Bank of Japan (DDBJ) and mirrored at the National Center for Biotechnology Information (NCBI) under BioProject accession numbers PRJDB6496 and PRJDB6504 (www.ncbi.nlm.nih.gov/bioproject/?term=PRJDB6496 and www.ncbi.nlm.nih.gov/bioproject/?term=PRJDB6504)

2. Metadata of all samples (such as individual sample accession numbers, information of multilocus sequence typing, geographical region, minimum inhibitory concentration values and genetic polymorphisms of each strain) is summarized in Tables S1 and S2 (available with the online version of this article).

**Impact Statement**

Antimicrobial resistance (AMR) is now recognized as one of the greatest threats to human health, and resistance in *Neisseria gonorrhoeae* is classified as one of the most urgent. The first extensively drug resistant strain with high resistance to first-line antimicrobial drugs was identified in 2009 in Japan, but subsequent surveillance has not been based on genome sequences. In this study, we conducted genomic surveillance and examined the AMR of >200 strains, as well as 67 additional strains from other time frames and locations within Japan. We discovered an interesting group of strains that had lost AMR due to the incorporation of DNA fragments from susceptible strains into a gene responsible for resistance. We also revealed the prevalence of strains resistant to each clinically important antimicrobial drug and investigated to what extent genetic resistance determinants can explain the observed phenotypic resistance. Furthermore, we revealed that the two major lineages spreading worldwide took very different evolutionary paths. Our study has major microbiological and clinical importance for the development of diagnostics and AMR tests, as well as for elucidating the origin and evolution of the resistant lineages that likely originated in Japan.

## Introduction

Antimicrobial resistance (AMR) is one of the greatest threats to human health and needs to be monitored and addressed at a global level [1, 2]. The number of infections caused by multidrug-resistant bacteria is increasing globally at a concerning rate [1]. Among bacterial pathogens that can be resistant to antimicrobial drugs, *Neisseria gonorrhoeae* is ranked as one of the three most ‘urgent’ threats by the CDC [3]. Gonorrhoea is one of the most common bacterial sexually transmitted infections worldwide and causes substantial morbidity and economic loss [4–6].

The first extensively drug resistant (XDR) gonorrhoea strain [7, 8] was H041, isolated in 2009 in Kyoto, Japan [9], followed by XDR strain F89 in Quimper, France [10]. XDR strains are defined as being resistant to two or more of the antibiotic classes generally recommended for the treatment of gonorrhoea, and three or more less frequently prescribed classes [7]. H041 and F89 showed comparatively high minimum inhibitory concentration (MIC) values of 2 and 1 µg ml^−1^, respectively [9, 10], to the antimicrobial drug ceftriaxone, which is currently the last remaining option for empirical first-line monotherapy in most countries. H041 and F89 were also resistant to fluoroquinolones and macrolides, including azithromycin, which together with ceftriaxone forms the dual therapy currently recommended for the treatment of gonorrhoea in international and national guidelines [6, 11, 12].

Large-scale whole-genome sequencing of bacterial isolates is quickly becoming the gold standard method for understanding AMR determinants and conducting surveillance of their prevalence and spread [13–17]. A recent genomic epidemiology study [18] analysed the genome sequences of 1102 gonococcal isolates sampled from across the USA over a 14 year period (2000–2013). The MIC values of extended-spectrum cephalosporins (ESCs, including cefixime and ceftriaxone), macrolides (azithromycin) and fluoroquinolones (ciprofloxacin) were examined to evaluate the extent to which known genetic resistance determinants can explain the observed phenotypic resistance [18]. Notably, this study reported the existence of unknown mechanisms of resistance to ceftriaxone and azithromycin, highlighting the importance of phenotypic surveillance.

However, such a genomic epidemiology study has not yet been conducted in the geographical regions where the first XDR strains were isolated. Although no other XDR strain has been subsequently reported in the local communities of Kyoto and Osaka, or elsewhere nationwide [19], Japan remains a region of global health concern for the emergence of XDR gonorrhoea bacteria due to high resistance rates to cefixime, azithromycin and ciprofloxacin [20]. It is, therefore, important to perform genomic surveillance to study the population structure of gonorrhoea bacteria in Japan at the genomic level, the presence of any lineage or sub-lineage that exhibits unusual patterns of reduced susceptibility to antimicrobials, and the evolution of such lineages. Additionally, the distribution of genetic resistance determinants and the extent to which these determinants can explain the observed phenotypic resistance in Japan remains unknown.

In this study, we explored these issues by analysing the whole-genome sequences and the antimicrobial-susceptibility testing results of 204 gonococcal strains sampled through genomic surveillance in the Japanese prefectures of Kyoto and Osaka (coloured in red on the map in Fig. S1) in 2015, as well as those of the H041 strain, as a reference. To provide context to our findings, we also analysed the whole-genome sequences of 67 additional gonococcal strains isolated from other time frames and locations within Japan. Our study revealed a detailed picture of the distribution of antimicrobial-resistance determinants and their relationship with phenotypic antimicrobial susceptibility and the evolution of two major lineages.

## Methods

### Isolates, antimicrobial susceptibility testing and DNA sequencing

Four outpatient clinics located in different areas (two in Kyoto and two in Osaka, Japan) provided specimens from all male patients who showed symptoms of gonorrhoea to the National Institute of Infectious Diseases (NIID), Tokyo, for the isolation of *N. gonorrhoeae*. Approximately 60 % of the specimens were culture-positive for *N. gonorrhoeae* (see [19] for details of the procedure), all of which were subject to antimicrobial-susceptibility testing and genome sequencing as follows: in 2015, a total of 204 *N. gonorrhoeae* isolates (1 per patient, from a total of 18 and 186 patients in Kyoto and Osaka, respectively) were obtained (Table S1), corresponding to approximately 23 % of male gonorrhoea patients reported by 65 sentinel clinics in Osaka in 2015, according to a public report of the Osaka prefecture. Although there could be potential biases in the patient population that is served by these clinics (e.g. socioeconomic biases, differences in the proportion of the population classified as men who have sex with men), this is the largest sampling of *N. gonorrhoeae* isolates conducted in the prefectures. The MICs (µg ml^−1^) of ceftriaxone, cefixime, ciprofloxacin and azithromycin were determined using the Etest method (bioMérieux) for each drug separately, according to the manufacturer’s instructions (Table S1). In order to define susceptible/intermediate/resistant phenotypes, we used the following MIC cut-offs according to the European Committee on Antimicrobial Susceptibility Testing (EUCAST; www.eucast.org/clinical_breakpoints): susceptibility ≤0.125 µg ml^−1^ and resistance >0.125 µg ml^−1^ for ceftriaxone and cefixime; susceptibility ≤0.25 µg ml^−1^ and resistance >0.5 µg ml^−1^ for azithromycin; and susceptibility ≤0.03 µg ml^−1^ and resistance >0.06 µg ml^−1^ for ciprofloxacin. Genomic DNA of each isolate was extracted with a MagNA Pure LC DNA isolation kit on a MagNA pure LC instrument (Roche Diagnostics), and was used for Nextera XT library construction and genome sequencing with an Illumina MiSeq 2×300 bp paired-end run protocol. The raw read data were deposited in the DNA Data Bank of Japan (DDBJ) and mirrored at the National Center for Biotechnology Information (NCBI) under BioProject accession number PRJDB6496. We measured the MICs using the Etest three times for the following strains for which the genotypes and phenotypes were initially discordant, and used median of MICs: FC488 (azithromycin), FC498 (ceftriaxone and cefixime), FC532 (ciprofloxacin) and FC524 (ciprofloxacin).

### Construction of the clonal phylogeny at the species level

The Illumina read data (300 bp paired-end reads) of each isolate were used for *de novo* assembly by A5-MiSeq with the default settings to produce contigs without scaffolding [21]. The number of contigs, N50 and raw coverage of each isolate are summarized in Table S1. We combined the data with the complete genome sequence of the H041 strain, which was recently determined using PacBio sequencing and named ‘WHO X’ [22]. Multilocus sequence typing (MLST) of the strains was conducted by exact string matching of the allelic sequences defined in PubMLST (www.pubmlst.org/neisseria) [23] to the contigs. If there was no exact string match, we conducted a blastn search of the contigs of the strain against the allelic sequences, and checked the top hit allele and its alignment. If the top hit had only a single nucleotide change, we assigned it to its closest sequence type (ST) and marked it with an asterisk (in the ST column in Table S1). We annotated each genome using Prokka [24]. As in a previous study [25], for single nucleotide polymorphism (SNP) calling and evolutionary analyses, we first conducted pairwise genome alignment between H041 (WHO X) and one of the 204 strains using progressiveMauve [26], rather than mapping their reads to the H041 (WHO X) reference genome. progressiveMauve enables the construction of positional homology alignments even for genomes with variable gene content and rearrangement. We then combined the alignments into a multiple whole-genome alignment, in which each position corresponded to that of the H041 (WHO X) genome. In the whole-genome alignment, we identified 24 455 core biallelic SNPs, which is comparable to the 34 959 core SNPs reported previously using a broader sampling: 242 gonococcal isolates collected by the CDC's Gonococcal Isolate Surveillance Project (GISP) from 28 cities in the USA in 2009–2010, which were included in a recent genomic epidemiology study [18] and have also been analysed separately [27]. For validation, we also constructed another whole-genome alignment using a pipeline [28] that similarly conducts pairwise genome alignment between a reference genome and one of the other genomes using MUMmer [29]. We confirmed that the genome contained a very similar number of core biallelic SNPs (22 239), in which 21 402 SNPs were shared between the two genome alignments. We constructed a maximum-likelihood tree using PhyML [30] from the former genome alignment containing the 24 186 core SNPs. We used the following parameters that indicate the GTR +G4 model of DNA substitution with estimation of the shape parameter of the gamma distribution by maximizing the likelihood: -m GTR -c 4 -a e. Using this as a starting tree, we constructed a clonal phylogeny at the species level with corrected branch lengths to account for homologous recombination using ClonalFrameML [31]. We first applied the standard model, and then applied the extended model using parameter estimates of the standard model: embranch_dispersion=0.1, kappa (relative rate of transitions vs transversions in substitution model)=3.148 as inferred by PhyML, initial value of R/θ (ratio of rates of recombination and mutation)=0.534, 1/δ (inverse mean DNA import length)=0.012, and ν (mean divergence of imported DNA)=0.094. The extended model allows for different recombination parameters to be inferred on different branches of the clonal phylogeny. Neither the maximum-likelihood starting tree, nor the clonal phylogeny, were rooted using an outgroup outside the species, and we showed the clonal phylogeny as mid-point rooted. We used the alignment sites that were present in at least 70 % of strains in order to focus on core and fairly conserved sites with a missing frequency ≤30 %. Furthermore, we confirmed that applying a cut-off of 90 % did not cause any visible change to the clonal phylogeny: the change in branch length was on average 1.6×10^−6^ nucleotide substitutions per site

### Analysis of genetic AMR determinants

For ESC (cefixime and ceftriaxone) resistance determinants, we extracted the nucleotide sequences of *penA*, which encodes the ESC-target penicillin-binding protein 2 (PBP2), by blastn searching the locus of the H041 (WHO X) strain against each of the other genomes. We used a blastn match of ≥70 % identity over ≥50 % of the locus length as the criteria for positive detection of the gene [32]. These criteria can extract both the mosaic and other *penA* alleles according to the sequence identities reported in previous studies [33, 34]. We constructed a neighbour-joining unrooted tree of *penA* using a Kimura two-parameter model implemented in mega 6 [35]. We used the entire coding sequence of *penA* in order to classify *penA* alleles according to their entire nucleotide sequences on the tree, which could then be compared to the clonal phylogeny. We also identified specific amino acid changes in PBP2 as well as PBP1 (encoded by *ponA*), the outer-membrane porin PorB (encoded by *penB*) and the MtrR efﬂux pump repressor, described in a recent review [36], using their amino acid sequences aligned using mafft v7.245 [37] and an in-house Perl script. We included the ESC-susceptible reference strain FA1090 (GenBank accession no. AE004969.1) in the alignment as a wild-type amino acid reference sequence, and examined the association between amino acid changes compared to the wild-type and resistance to cefixime by Fisher’s exact test and multiple testing correction via false discovery rate (FDR) [38] implemented in R version 3.3.1. For fluoroquinolone (ciprofloxacin) resistance determinants, we examined nonsynonymous substitutions in *gyrA* and *parC* described in a recent review [36]. For macrolide (azithromycin) resistance determinants, we examined the two known primary mechanisms [39]: adenosine deletion in the promoter region of *mtrR* [36], and the A2059G and C2611T (*Escherichia coli* numbering) mutations in the 23S rRNA gene [40]. We detected the presence or absence of these variants using ariba, which uses a combined mapping/alignment and targeted local assembly approach [41]. For macrolide (azithromycin) resistance determinants, we also looked for the presence or absence of *erm* genes [39] and the premature stop codon in the coding sequence of *mtrR* (e.g. allele 367 defined in the PubMLST *Neisseria* database [36, 42]), although none were found among the 204 isolates and we excluded them from subsequent analyses. For plasmid-encoded resistance genes, we also looked for the presence or absence of the *bla_TEM_* and *tetM* genes [22, 36] using a blastn match of ≥70 % identity over ≥50 % of the locus length. The presence or absence of these genetic AMR determinants as well as the MICs of the antimicrobial drugs for each strain were illustrated as heatmaps using Phandango [43].

### Inference of recombination in the susceptible reverted *penA* allele

To confirm a recombination event (DNA import) from an external source into the susceptible reversed *penA* allele of a ST7363 sub-lineage, we constructed a subset of the multiple whole-genome alignment described above for the strains in the sub-lineage using H041 (WHO X) as a reference and the FC489 strain carrying the *penA* X allele as an outgroup. We selected strain FC489 as the outgroup because it was not included in the sub-lineage but was its nearest neighbour: sequence identity calculated from the whole-genome alignment excluding gapped sites between the outgroup strain FC489 and strain KM304 in the sub-lineage is 99.95 %, equal to the lowest sequence identity between strains within the sub-lineage. We then constructed a maximum-likelihood tree using PhyML [30] and conducted a recombination analysis using Gubbins [44] by specifying the maximum-likelihood tree as a starting tree and strain FC489 as an outgroup. We then conducted a blastn search of a recombined fragment overlapping *penA* in all 204 *N. gonorrhoeae* genomes in order to explore potential recombination sources.

### Additional dataset of ST7363 and ST1901 strains

To compare our findings with those of other time points and geographical regions, we selected the following isolates and conducted genome sequencing of our isolate collection (Table S2) according to the Illumina MiSeq procedure described previously. First, we searched for and selected 40 ST7363 strains (out of 272 strains in total) isolated in other geographical regions in 2015. Specifically, they were isolated in Miyagi, Saitama, Aichi and Gifu, Japan (coloured in blue on the map in Fig. S1). Second, we looked for ST7363 strains with cefixime MICs<0.016 µg ml^−1^ (the lowest value measured by Etest as most likely indicating the presence of a susceptible *penA* allele) isolated in Kyoto and Osaka during 2011–2014 and selected six isolates that fulfilled the criteria. Third, we looked for ST7363 and ST1901 strains that were isolated in the 1990s in a different geographical region to be used as outgroups for Bayesian evolutionary analyses of the two lineages. From amongst such historical strains isolated in Kanagawa, Japan (coloured in orange on the map in Fig. S1), we selected 1 of 6 ST7363 strains isolated in 1996, whereas we selected all 20 ST1901 strains isolated from 1996 to 2000. We selected the larger number of ST1901 strains based on preliminary analyses in which we added a single or a few such strains for construction of time-resolved phylogeny of ST1901 (next section) but did not obtain an interpretable phylogeny carrying a particular root. The MICs (µg ml^−1^) of ceftriaxone and cefixime of the historical strains isolated in Kanagawa were determined by agar-dilution at the Kanagawa Prefectural Institute of Public Health. The raw read data were deposited in the DDBJ and mirrored at the NCBI under BioProject accession number PRJDB6504.

### Construction of time-resolved phylogeny of the two major lineages

We conducted pairwise genome alignment between H041 (WHO X) and each of the additional ST7363 and ST1901 strains using the same methods as above. We then combined them with a subset of the multiple whole-genome alignment described above for the strains in the ST7363- and ST1901-associated lineages, separately. From the whole-genome alignment of each lineage, we constructed a maximum-likelihood tree using PhyML with the same model and parameters described above. In order to demonstrate that the data of the lineages have temporal signal, we plotted the sampling year versus root-to-tips correlation using TempEst [45] (Fig. S2). We also performed the date randomization tests using the ‘roottotip’ function of BactDating [46] and confirmed that the temporal signal is significant in both ST7363- and ST1901-associated lineages (*P*=0.018 and *P* <10^−5^). Using the maximum-likelihood tree as a starting tree, we applied the standard model of ClonalFrameML to each linage and inferred individual recombination events imported from outside the lineage. We excluded all recombined sequences from the whole-genome alignment, and then extracted 1079 and 3255 core biallelic SNPs for the ST7363- and ST1901-associated lineages. We then constructed a time-resolved phylogeny using the SNPs and beast version 1.10.0 [47], for which we specified the GTR +G4 model of DNA substitution with estimation of the shape parameter of the gamma distribution. We estimated and compared log marginal likelihood of the constant population size model and the exponential population growth model using generalized stepping-stone sampling (GSS) [48]. beast was run for 100 million Markov chain Monte Carlo (MCMC) iterations, followed by 100 path steps of 1 million iterations for GSS, with samples taken every 1000 iterations. We used Tracer v1.7 [49] to check that the effective sample size (ESS) of all parameters exceeded 500 and that runs had converged. We chose the simpler constant population size model because it showed a larger log marginal likelihood (Bayes Factor >3.5) for both the ST7363- and ST1901-associated lineages. We also tried to apply the relaxed clock rate model coupled with the constant population size model, but did not choose it because it yielded ESS <200 for several parameters. The time-resolved phylogeny was automatically rooted by beast using outgroup strains in each lineage. We prepared the input xml files based on those exported from BEAUti version 1.8.4 rather than version 1.10.0, because the latter caused runtime errors in GSS, and made them publicly available at https://figshare.com/s/1f12166596282b841e05. A maximum clade credibility tree was generated for ST7363 and ST1901 after discarding 10 % as burn-in by using TreeAnnotator (part of the beast package). All nodes and branches with posterior support values with ≥ 0.9 were marked using FigTree version 1.4.3.

## Results

### Analysis of susceptibility to the ESCs, examination of *penA* alleles and the discovery of a novel sub-lineage

**Fig. 1.**
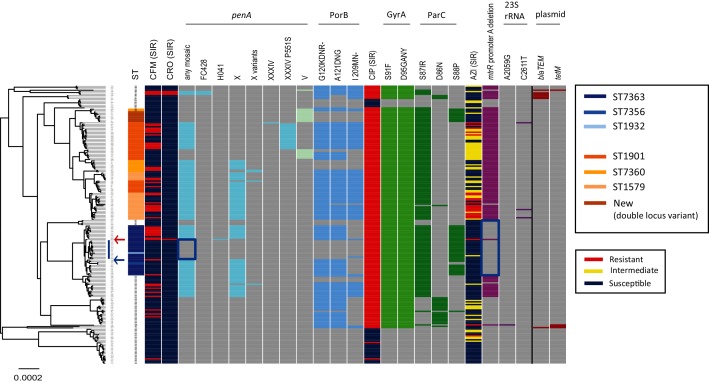
Whole-genome sequence phylogeny, resistance patterns of the antimicrobials and genetic polymorphisms. Left: a clonal phylogeny with corrected branch lengths to account for homologous recombination. The blue vertical line represents the notable sub-lineage in ST7363, whereas the blue arrow represents the outgroup of the sub-lineage. The red arrow indicates strain H041 (WHO X). Heatmap: column 1, ST: the two major STs (ST7363 and ST1901), single-locus variants of the former (ST7356, ST1932) and the latter (ST7360 and ST1579), and a new ST (double-locus variant of ST1901) are coloured. Columns 2 and 3: susceptible/resistant (S/R) categories according to the EUCAST MIC breakpoint of cefixime (CFM) and ceftriaxone (CRO). The presence or absence of genetic features is shown in columns 4–14, 16–20, 22–26 as marked: grey indicates absence, other colours indicate presence. Column 4: any mosaic *penA* allele. Columns 5–10: a specific mosaic *penA* allele. Column 11: *penA* V. Columns 12–14: nonsynonymous amino acid changes from wild-types in PorB. Column 15: susceptible/intermediate/resistant (S/I/R) categories according to the EUCAST MIC breakpoints of ciprofloxacin (CIP). Columns 16–17: nonsynonymous amino acid changes from wild-type in GyrA. Columns 18–20: nonsynonymous amino acid changes from wild-type in ParC. Column 21: susceptible/intermediate/resistant (S/I/R) categories according to the EUCAST MIC breakpoints of azithromycin (AZI). Column 22: the adenosine deletion in the *mtrR* promoter. Columns 23–24: the mutations in the 23S rRNA gene. Columns 25–26: plasmid-encoded *bla_TEM_* and *tetM*. The blue rectangles indicate notable sub-lineages in ST7363 described in the text.

For the 204 strains sampled in the Japanese prefectures of Kyoto and Osaka in 2015 (Table S1) and reference strain H041 (WHO X), a clonal phylogeny with branch lengths corrected to account for homologous recombination was inferred using ClonalFrameML (Fig. 1). The ratio of rates of recombination and mutation was estimated to be 0.56, whereas the ratio of the number of substitutions predicted to have been imported through recombination and point mutation was 5.94, a relatively high value among bacterial species [50].

The two major STs, ST7363 (36 isolates) and ST1901 (33 isolates), and their variants are highlighted in the first column of Fig. 1. In total, 20 STs were found, and their frequency distribution is shown in Fig. S3. Susceptible/resistant categories according to the EUCAST MIC breakpoint (susceptibility ≤0.125 µg ml^−1^ and resistance >0.125 µg ml^−1^) of ESCs (cefixime and ceftriaxone) are coloured in the second and third columns in Fig. 1. The presence or absence of any mosaic *penA* allele (specifically, FC428-type [34], H041-type [9], X and its variants [19], and XXXIV and its variant [19] in this paper) is indicated in the fourth column. The *penA* alleles have been designated according to amino acid sequences encoded by the whole gene, and alleles such as X and XXXIV were termed ‘mosaic’ because the second half of their DNA sequences appear to have been imported from other *Neisseria* spp. that are naturally resistant to ESCs through homologous recombination events [51]. The presence or absence of a specific *penA* allele is indicated in the 5th–11th columns. The 11th column corresponds to *penA* allele V, which is not one of the mosaic alleles but confers susceptibility to ESCs. The most frequent mosaic *penA* alleles were X and its variants, whereas a previous study in the USA focused on *penA* XXXIV and its variants, as they were predominant in the sampled time frame (2005–2013) [18].

The MIC values of cefixime and ceftriaxone were significantly higher in the presence of the mosaic *penA* alleles (Figs S4 and S5, *P*<10^−15^, Wilcoxon’s rank-sum test). However, aside from H041 (WHO X), only three strains (FC498, FC460 and FC428) satisfied the ceftriaxone-resistance cut-off (MIC>0.125 µg ml^−1^), all of which carried FC428-type *penA* [34]. There were 51 strains satisfying the cefixime-resistance cut-off (MIC>0.125 µg ml^−1^) that were associated with the mosaic *penA* with 92 % sensitivity and 62 % specificity (Fig. S5).

We also examined the specific amino acid changes of PBP2 (encoded by *penA*), as well as PBP1 (encoded by *ponA*), the outer-membrane porin PorB (encoded by *penB*) and the MtrR efﬂux pump repressor. Among 217 sites that had amino acid changes, 51 sites showed a significant association with cefixime resistance (*P_FDR_* <10^−5^, Fisher’s exact test), consisting of 48 sites in PBP2 and 3 sites in PorB. The most significant association (*P*=3×10^−12^, *P_FDR_*=5×10^−11^) was found in PBP2 N513Y and G546S, and their sensitivity (92 %) and specificity (62 %) were the same as those of mosaic *penA*. These findings are expected since the amino acids are located in the C-terminal domain of PBP2, characteristic of mosaic *penA* [9]. Among the 48 significant sites in PBP2, 42 were located in the C-terminal domain (sites 279, 288, 291, 312, 316, 323, 326, 328, 329, 330, 331, 332, 335, 341, 342, 343, 345, 353, 374, 376, 377, 378, 386, 389, 407, 412, 413, 438, 444, 448, 458, 462, 463, 465, 469, 470, 473, 481, 513, 517, 542 and 546), whereas 6 were in the N-terminal domain (160, 173, 202, 203, 204 and 214). Regarding PorB, the presence or absence of the amino acid changes in the three significant sites (120, 121 and 210) is indicated in the 12th–14th columns in Fig. 1. The amino acid changes in PorB were significantly associated with mosaic *penA* (*P*<5×10^−15^), suggesting an epistatic interaction between them >270 kb apart in the chromosome.

ST7363 (coloured blue in the ST column in Fig. 1) formed a single lineage in the phylogeny, which contained only ST7363 and its single locus variants (ST7356 or ST1932). Interestingly, however, the lineage was divided into two sub-lineages: one included the H041 (WHO X) strain and contained mosaic *penA* alleles (H041-type or X), while the other (blue rectangle in the any mosaic column in Fig. 1) did not have any mosaic *penA* alleles and was susceptible to cefixime and ceftriaxone.

**Fig. 2.**
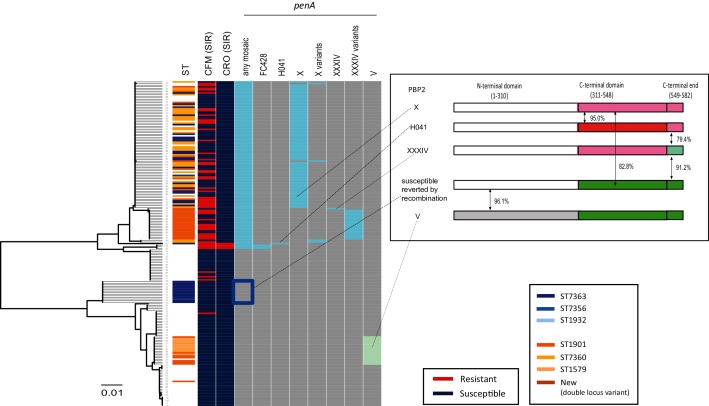
Gene tree of *penA*, resistance patterns of cefixime and ceftriaxone, and *penA* polymorphisms. Heatmap: column 1, ST: the ST of the genomes harbouring the *penA* alleles. The two major STs (ST7363 and ST1901), single-locus variants of the former (ST7356, ST1932) and the latter (ST7360 and ST1579), and a new ST (double-locus variant of ST1901) are coloured. Columns 2–3: susceptible/resistant (S/R) categories according to the EUCAST MIC breakpoint of cefixime (CFM) and ceftriaxone (CRO). The presence or absence of genetic features is shown in columns 4–11 as marked: grey indicates absence, other colours indicate presence. Column 4: any mosaic *penA* allele. Columns 5–10: a specific mosaic *penA* allele. Column 11: *penA* V. The inset on the right is a schematic representation illustrating PBP2 amino acid sequences encoded by the *penA* alleles. The sequence blocks shaded with the same colour represent identical amino acid sequences. The susceptible *penA* allele reverted by recombination is composed of *penA* X (first half, white) and *penA* V (second half, yellow-green).

A neighbour-joining tree of *penA* nucleotide sequences is shown in Fig. 2. The mosaic and other *penA* alleles were completely separated into two clusters (upper and lower) in the tree. The mosaic *penA* alleles were carried by the majority of the ST1901 and ST7363 strains; exceptions were the susceptible sub-lineage of ST7363 (indicated by the blue rectangle in the any mosaic column in Fig. 2) and the sub-lineage of ST1901 that carried the *penA* V allele. Examination of the susceptible *penA* allele in the sub-lineage of ST7363 revealed that the first half of the sequence was identical to that of the mosaic *penA* X or H041 alleles, but that the second half was recombined with that of the *penA* V allele (inset in Fig. 2).

The susceptible sub-lineage of ST7363 seems to have evolved from its ancestor, which carried *penA* X (Fig. 1). Genome sequences within the sub-lineage are very closely related (≥99.97 % sequence identity). Inference of individual recombination events that imported DNA fragments from external sources into the sub-lineage using Gubbins revealed a recombined fragment spanning approximately 3.3 kb from the second half of *penA* to the 2.5 kb downstream intergenic region. A blastn search of the recombined fragment in the genomic population of the 204 strains confirmed that a strain carrying *penA* V was a donor for recombination, evident because the whole fragment had >99.98 % sequence identity with strains carrying *penA* V. The next most closely related blastn hit was 99.34 %, found in strain KM311 (lower in Fig. 1, outside the ST7363- and ST1901-associated linages) that encodes a 2 amino acid variant of *penA V*. We do not term this allele, derived from *penA* X and *penA* V, mosaic because it indicates an allele that can confer resistance to ESCs, which is not the case for this allele.

**Fig. 3.**
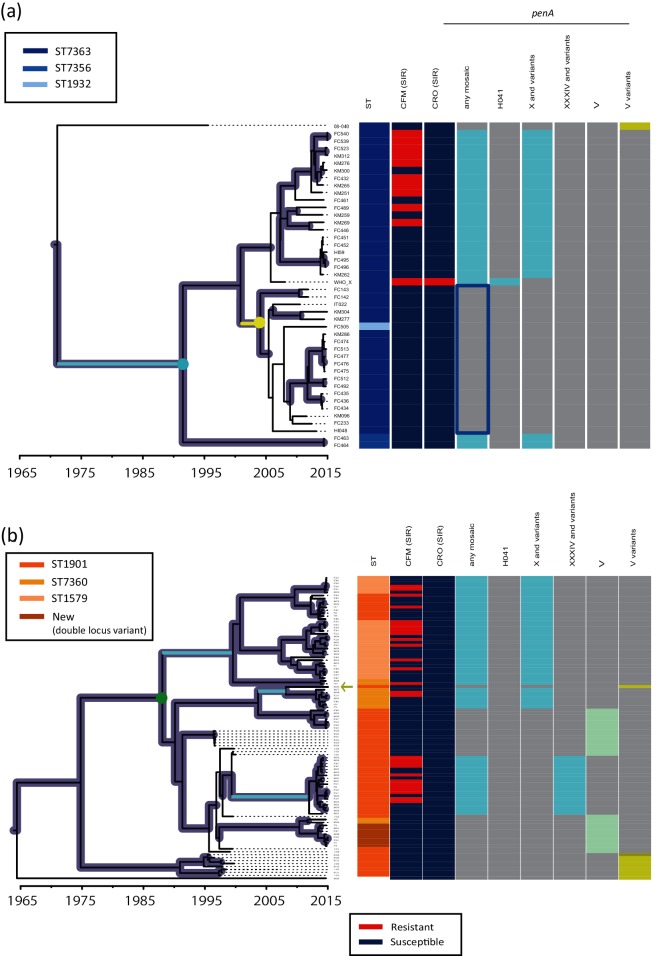
Time-resolved phylogeny and *penA* polymorphisms. All nodes and branches with posterior support values with 0.9 are coloured in purple. The cyan branches indicate acquisition of *penA* X or XXXIV, whereas the yellow branch indicates loss of *penA* X by recombination. The cyan, yellow and green circles indicate nodes with 95 % HPD described in the text. Heatmap: column 1, ST: the ST of the genomes harbouring the *penA* alleles. The two major STs (ST7363 and ST1901), single-locus variants of the former (ST7356, ST1932) and the latter (ST7360 and ST1579), and a new ST (double-locus variant of ST1901) are coloured. Columns 2–3: susceptible/resistant (S/R) categories according to the EUCAST MIC breakpoint of cefixime (CFM) and ceftriaxone (CRO). The presence or absence of genetic features is shown in columns 4–9 as marked: grey indicates absence, other colours indicate presence. Column 4: any mosaic *penA* allele. Columns 5–7: *penA* H041-type, X and variants, and XXXIV and variants. Column 8: *penA* V. Column 9: variants of *penA* V: light and dark yellow-green indicate 4 and 5 amino acid variants, respectively. (a) ST7363 and its single locus variants. The blue rectangle indicates the notable sub-lineage sharing the susceptible *penA* allele reverted by recombination. (b) ST1901-associated lineage. The yellow-green arrow indicates an exceptional strain isolated in 2015 that contained the ancestral susceptible *penA* V variant.

Metadata of each isolate, such as MLST information (including allele numbers of the seven loci), MIC values and genetic polymorphisms are summarized in Table S1. Amino acid sequences of the *penA* alleles in Fig. 2, *penA* V variants in Fig. 3 and the ESC-susceptible reference strain FA1090 are summarized as a supplementary fasta file (https://figshare.com/articles/penA_sequences/6756845).

### Quinolone resistance

Approximately 85 % of the strains showed resistance to fluoroquinolones (ciprofloxacin MIC >0.06 µg ml^−1^ according to EUCAST). In *N. gonorrhoeae*, quinolone resistance has been attributed to nonsynonymous substitutions in specific regions of *gyrA* and *parC*, namely GyrA amino acid positions 91 and 95, and ParC position 87, and less frequently ParC positions 86 and 88 [18]. Susceptible/resistant categories of ciprofloxacin and the presence of these substitutions are summarized in the 15th–20th columns in Fig. 1. The resistance is explained by completely linked amino acid substitutions at positions 91 and 95 of GyrA with 99 % sensitivity and 100 % specificity. Only two of the resistance strains did not have the linked amino acid substitutions (Fig. S6). Among the *parC* polymorphisms, ParC S87IR was the most frequent. Most strains carried either ParC D86N or ParC S87IR, while no strain carried both. Additionally, all strains carrying *parC* S88P also had *parC* S87IR. Strains without the amino acid substitutions in GyrA formed two groups in the clonal phylogeny (upper and bottom in Fig. 1). They had none of the substitutions attributed to quinolone resistance in GyrA or ParC.

### Azithromycin resistance

Approximately 10 % of the strains showed resistance to azithromycin (MIC >0.5 µg ml^−1^ according to EUCAST). The resistance has previously been attributed to mutations in the 23S rRNA gene or in the *mtrR* (a repressor of efflux pump) locus or its promoters [18, 40]. In our dataset, the resistance is explained by an adenosine deletion in the promoter region of *mtrR*, with 95 % sensitivity, but only 52 % specificity (Fig. S7). Susceptible/intermediate/resistant categories and the presence of the deletion as well as of the mutations (C2611T or A2059G) in the 23S rRNA gene are summarized on the right-hand side of Fig. 1. All ST1901 strains had the deletion (Fig. 1). In contrast, the deletion in the promoter region of *mtrR* was not found in any ST7363 strains except for H041 (WHO X) (blue rectangle in the *mtrR* promoter A deletion column in Fig. 1). Further examination of another dataset of 40 ST7363 strains isolated in 2015 in other geographical regions (Miyagi, Saitama, Aichi and Gifu, Japan, coloured in blue in Fig. S1) (Table S2) revealed an almost perfectly consistent result, with 93 % of the strains not harbouring the adenosine deletion in the *mtrR* promoter.

The A2059G and C2611T mutations in the 23S rRNA gene were found only in a single strain (FC488), which showed high-level resistance, with an azithromycin MIC of >256 µg ml^−1^, and the three strains with MIC values of 16, 8 and 1 [39] (Table S1). The three showing an azithromycin MIC >1 were the only strains that carried one of the mutations in the 23S rRNA gene. To account for the presence of four copies of the gene in the *N. gonorrhoeae* genome, we examined the depth of reads mapped to each nucleotide at the two positions in the four strains. In the strain carrying A2059G, all reads covering that position were mapped to the resistant nucleotide. In two out of the three strains carrying C2611T with MIC values of 8 and 16, all or almost all (>99 %) reads covering that position were mapped to the resistant nucleotide, whereas only 60 % of reads did so in the remaining strain that had a MIC value of 1.

### Plasmid-encoded resistance

The *bla_TEM_* gene, which confers resistance to β-lactams, was not found in either ST7363- or ST1901-associated clades. Rather, it was found in ST7827, ST1903 (upper in Fig. 1), ST1599 and ST13476 (bottom in Fig. 1). Similarly, *tetM*, which confers resistance to tetracycline, was found only in ST1599 (upper in Fig. 1), ST10899, ST1597 and ST13476 (lower in Fig. 1). Only two strains (FC493 of ST1599 and KM311 of ST13476) were found to carry both the genes, each of which was originally located in different plasmids [22, 36]. Although it is challenging to reconstruct plasmids themselves from the Illumina short-read data, the results show that the plasmid-encoded resistance in this geographical region is confined to the minor STs that consist of the two lineages outside ST7363 and ST1901.

### Evolution of ST7363- and ST1901-associated lineages and *penA* alleles

For further examination of the susceptible *penA* allele reverted by recombination and the sub-lineage in ST7363, we investigated *penA* alleles of six historical ST7363 strains with cefixime MICs <0.016 µg ml^−1^ (the lowest value measured by Etest, most likely indicating presence of a susceptible *penA* allele) isolated in Kyoto and Osaka during 2011–2014 (Table S2). We have confirmed that all strains carried the susceptible reverted allele. Next, we constructed a time-resolved phylogeny of the ST7363-associated lineage (Fig. 3a) by adding one historical ST7363 strain isolated in Kanagawa (coloured in orange in Fig. S1) in 1996 in addition to the six historical strains. The phylogeny clearly shows that *penA* evolved from an ancestral susceptible allele (4 amino acid variant of *penA* V) to *penA* X by a single event (on a cyan branch in Fig. 3a) prior to 1998 [at the node marked by a cyan circle in with 95 % highest posterior density (HPD) (1984.0–1997.8)]. The susceptible allele reverted by recombination between *penA* X and *penA* V, emerged by a single event (on a yellow branch in Fig. 3a) prior to 2007 [at the node marked by a yellow circle in with 95 % HPD (2001.2–2006.5)], and have been inherited and shared among the sub-lineage and ancestors (blue rectangle in Fig. 3a).

By contrast, ST1901 (coloured in orange in ST column in Fig. 1) and its variants (single locus variants ST7360 and ST1579 and a new ST, which is a double locus variant of ST1901, coloured in light orange) formed a large lineage in which ST1901 seemed to be paraphyletic, corresponding to different *penA* alleles: X and its variants, XXXIV and its variant, and V. In other words, the two sub-lineages carrying the two types of mosaic *penA* alleles (X and XXXIV) are phylogenetically distinct at the core genome level even in this single geographical region. In order to understand how they evolved, we constructed a time-resolved phylogeny of the ST1901-associated lineage (Fig. 3b) by adding 20 historical ST1901 strains isolated in Kanagawa from 1996 to 2000. The time-resolved phylogeny shows *penA* evolved from the ancestral susceptible allele (a 4 amino acid variant of *penA* V) as that of ST7363. Before the 1990s [i.e. at the node marked by a green circle in Fig. 3(b) with 95 % HPD (1985.8–1989.0)], the ancestral allele was replaced by *penA* V. After that, the mosaic *penA* X and XXXIV were likely to have been separately acquired by their ancestors. In particular, *penA* X was likely to have been acquired twice on different branches. Meanwhile, the sub-lineages of ST1901 carrying *penA* V were not completely replaced by those carrying *penA* X or XXXIV, but were maintained at low frequency. Exceptionally, a singleton strain KM309 isolated in Osaka in 2015 (yellow-green arrow in Fig. 3b, next to the strains carrying *penA* X) carried the ancestral susceptible *penA* (the 4 amino acid variant of *penA* V), which is likely to have been reverted from *penA* X by recombination.

## Discussion

We conducted genomic surveillance in 2015 and a genomic epidemiology study in a Japanese region where the first isolate of XDR ceftriaxone-resistant *N. gonorrhoeae* was identified. We confirmed that no additional XDR ceftriaxone-resistant strains have emerged thus far, similar to other regions in the world, potentially indicating that XDR strains suffer from decreased biological fitness [52]. Mutations in *penA* that confer resistance to ESCs are predicted to have a negative impact on gonococcal fitness, given that this gene functions in cell-wall biosynthesis [53].

Despite finding no additional XDR ceftriaxone-resistant strains, we discovered a sub-lineage of ST7363 that had lost the mosaic *penA* allele responsible for reduced susceptibility to ESCs, and instead maintained a susceptible *penA* allele that had been reversed by recombination. We also discovered that none of the ST7363 strains, except for H041 (WHO X), harboured the adenosine deletion in the *mtrR* promoter, which predicts azithromycin resistance with 95 % sensitivity (but only 52 % specificity). A recent study examining 75 azithromycin-resistant *N. gonorrhoeae* isolates cultured from 2009 to 2014 in 17 European countries did not find an ST7363 isolate without the deletion [40]. Although it has been suggested that most AMR mechanisms in *N. gonorrhoeae* do not appear to confer a significant fitness cost [52], these findings suggest that loss of resistance determinants could be advantageous for gonococci, depending upon environmental conditions. Otherwise, the loss of resistance could be due to sufficient recombination and a lack of selection for resistance. The availability of regional information regarding antimicrobial usage would be helpful to formally quantify the fitness costs and benefits of resistance [54], although no such data are currently accessible.

A similar re-emergence of antimicrobial susceptibility in a resistant lineage was recently reported in *N. gonorrhoeae* in the USA [17, 18], as well as in *Staphylococcus aureus* in the UK [55]. The USA study reported that the strains susceptible to ESCs appear to have undergone a recombination event that replaced the resistant mosaic *penA* XXXIV allele with the susceptible mosaic *penA* XXXVIII. Similarly, we were able to reconstruct the evolutionary history of the sub-lineage as follows: a DNA fragment was imported from outside the lineage into the second half of the *penA* locus, resulting in creation of the susceptible reverted allele; the donor of recombination was identified as *penA* V. Whilst previous studies have not examined MLST information, we carefully examined the relationships between ST and sub-lineage.

We found that most (approximately 85 %) of the isolates in the Japanese region (Kyoto and Osaka) were quinolone resistant. This finding is consistent with previous observations that most quinolone-resistance mutations in *N. gonorrhoeae* are associated with little to no ﬁtness cost [18]. Otherwise, there is also a possibility that the strains harbour compensatory mutations to recover fitness costs, as recently reported for mosaic *penA* alleles [53]. Recent studies in China [56, 57] showed that the frequency of quinolone-resistant strains was almost 100 % in 2008, 2010 and 2012–13. These observations could be due to heavy quinolone use in these countries. In addition, the simultaneous amino acid substitutions in GyrA 91 and 95 could confer an *in vivo* fitness benefit to the wild-type, as has been demonstrated in a competition experiment [58].

Our genomic surveillance revealed a detailed picture of the distribution of AMR determinants and their relation to phenotypic antimicrobial susceptibility in a region of public health concern. The results particularly suggest that the simultaneous amino acid substitutions in GyrA 91 and 95 could be a marker of resistance to fluoroquinolones in this geographical region, in addition to other geographical regions such as the USA [17], Russia [59] and India [60] where a strong association between the substitutions in GyrA and fluoroquinolone resistance has been reported. The marker can be utilized for point-of-care diagnostic and AMR tests (POC-AMR), the importance of which has been shown for enabling prompt diagnosis and individualized treatment, and helping to combat the spread of AMR [61, 62]. Such tests are already in development: for example, nucleic acid amplification tests (NAATs) detect markers in *gyrA* directly from clinical samples [63]. Our study revealed that there was no single marker capable of discriminating reduced susceptibility to ESCs and azithromycin with sufficient sensitivity and specificity, suggesting the importance of additional (perhaps combinatorial) markers.

The dataset analysed in the present study is publicly available as a BioProject deposited in DDBJ/NCBI (Table S1). To our knowledge, this is the largest dataset that consists of both raw read sequences and MIC values in response to ESCs, fluoroquinolone and macrolides of more than 200 strains sampled in a single geographical region. We expect that the dataset will be a valuable resource for future studies, in particular for exploring additional markers for resistance.

Furthermore, by combining the dataset with that of the historical gonococcal strains within Japan, we were able to reveal the different evolutionary paths of the two major lineages that have been spreading worldwide that likely originated in Japan [64]. The ST7363-associated lineage evolved from a susceptible ancestor that acquired *penA* X, and underwent recombination between *penA* X and *penA* V in the sub-lineage. By contrast, the evolution of the ST1901-associated lineage was more complicated: although the ancestral susceptible *penA* allele was the same as that of the ST7363-associated lineage, *penA* X and XXXIV were acquired independently on different ancestral lineages. Our study provides a solid basis for the further elucidation of the origin and evolution of the two major lineages that are spreading worldwide.

## Data bibliography

1. Yahara, Nakayama, Shimuta, Lee, Ohnishi *et al.* DDBJ. PRJDB6496 (2018).
2. Yahara, Nakayama, Shimuta, Lee, Ohnishi *et al.* DDBJ. PRJDB6504 (2018).

## Supplementary Data

Supplementary File 1

Supplementary File 2

## References

[1] Blair JM, Webber MA, Baylay AJ, Ogbolu DO, Piddock LJ (2015). Molecular mechanisms of antibiotic resistance. Nat Rev Microbiol.

[2] Sugden R, Kelly R, Davies S (2016). Combatting antimicrobial resistance globally. Nat Microbiol.

[3] CDC (2013). Antibiotic Resistance Threats in the United States..

[4] Newman L, Rowley J, vander Hoorn S, Wijesooriya NS, Unemo M (2015). Global estimates of the prevalence and incidence of four curable sexually transmitted infections in 2012 based on systematic review and global reporting. PLoS One.

[5] WHO (2008). Global Incidence and Prevalence of Selected Curable Sexually Transmitted Infections.

[6] WHO (2012). Global Action Plan to Control the Spread and Impact of Antimicrobial Resistance in Neisseria gonorrhoeae.

[7] Tapsall JW, Ndowa F, Lewis DA, Unemo M (2009). Meeting the public health challenge of multidrug- and extensively drug-resistant *Neisseria gonorrhoeae*. Expert Rev Anti Infect Ther.

[8] Goire N, Lahra MM, Chen M, Donovan B, Fairley CK (2014). Molecular approaches to enhance surveillance of gonococcal antimicrobial resistance. Nat Rev Microbiol.

[9] Ohnishi M, Golparian D, Shimuta K, Saika T, Hoshina S (2011). Is *Neisseria gonorrhoeae* initiating a future era of untreatable gonorrhea?: detailed characterization of the first strain with high-level resistance to ceftriaxone. Antimicrob Agents Chemother.

[10] Unemo M, Golparian D, Nicholas R, Ohnishi M, Gallay A (2012). High-level cefixime- and ceftriaxone-resistant *Neisseria gonorrhoeae* in France: novel penA mosaic allele in a successful international clone causes treatment failure. Antimicrob Agents Chemother.

[11] Bignell C, Unemo M, Radcliffe K, Jensen JS, Babayan K (2013). 2012 European guideline on the diagnosis and treatment of gonorrhoea in adults. Int J STD AIDS.

[12] Workowski KA, Bolan GA (2015). Sexually transmitted diseases treatment guidelines, 2015. MMWR Recomm Rep.

[13] de Silva D, Peters J, Cole K, Cole MJ, Cresswell F (2016). Whole-genome sequencing to determine transmission of *Neisseria gonorrhoeae*: an observational study. Lancet Infect Dis.

[14] Ronholm J, Nasheri N, Petronella N, Pagotto F (2016). Navigating microbiological food safety in the era of whole-genome sequencing. Clin Microbiol Rev.

[15] Allen VG, Melano RG (2016). Whole-genome sequencing-new tools for gonorrhoea control. Lancet Infect Dis.

[16] Ohnishi M, Unemo M (2014). Phylogenomics for drug-resistant *Neisseria gonorrhoeae*. Lancet Infect Dis.

[17] Grad YH, Kirkcaldy RD, Trees D, Dordel J, Harris SR (2014). Genomic epidemiology of *Neisseria gonorrhoeae* with reduced susceptibility to cefixime in the USA: a retrospective observational study. Lancet Infect Dis.

[18] Grad YH, Harris SR, Kirkcaldy RD, Green AG, Marks DS (2016). Genomic epidemiology of gonococcal resistance to extended-spectrum cephalosporins, macrolides, and fluoroquinolones in the United States, 2000–2013. J Infect Dis.

[19] Shimuta K, Unemo M, Nakayama S, Morita-Ishihara T, Dorin M (2013). Antimicrobial resistance and molecular typing of *Neisseria gonorrhoeae* isolates in Kyoto and Osaka, Japan, 2010 to 2012: intensified surveillance after identification of the first strain (H041) with high-level ceftriaxone resistance. Antimicrob Agents Chemother.

[20] Wi T, Lahra MM, Ndowa F, Bala M, Dillon JR (2017). Antimicrobial resistance in *Neisseria gonorrhoeae*: global surveillance and a call for international collaborative action. PLoS Med.

[21] Coil D, Jospin G, Darling AE (2015). A5-miseq: an updated pipeline to assemble microbial genomes from Illumina MiSeq data. Bioinformatics.

[22] Unemo M, Golparian D, Sánchez-Busó L, Grad Y, Jacobsson S (2016). The novel 2016 WHO *Neisseria gonorrhoeae* reference strains for global quality assurance of laboratory investigations: phenotypic, genetic and reference genome characterization. J Antimicrob Chemother.

[23] Maiden MC, Harrison OB (2016). Population and functional genomics of *Neisseria* revealed with gene-by-gene approaches. J Clin Microbiol.

[24] Seemann T (2014). Prokka: rapid prokaryotic genome annotation. Bioinformatics.

[25] Zhang G, Leclercq SO, Tian J, Wang C, Yahara K (2017). A new subclass of intrinsic aminoglycoside nucleotidyltransferases, ANT(3")-II, is horizontally transferred among *Acinetobacter* spp. by homologous recombination. PLoS Genet.

[26] Darling AE, Mau B, Perna NT (2010). progressiveMauve: multiple genome alignment with gene gain, loss and rearrangement. PLoS One.

[27] Grad YH, Kirkcaldy R, Trees D, Dordel J, Goldstein E (2013). O03.4 Genomic epidemiology of *Neisseria gonorrhoeae* with reduced susceptibility to cefixime in the United States. Sex Transm Infect.

[28] Didelot X, Pang B, Zhou Z, McCann A, Ni P (2015). The role of China in the global spread of the current cholera pandemic. PLoS Genet.

[29] Kurtz S, Phillippy A, Delcher AL, Smoot M, Shumway M (2004). Versatile and open software for comparing large genomes. Genome Biol.

[30] Guindon S, Dufayard JF, Lefort V, Anisimova M, Hordijk W (2010). New algorithms and methods to estimate maximum-likelihood phylogenies: assessing the performance of PhyML 3.0. Syst Biol.

[31] Didelot X, Wilson DJ (2015). ClonalFrameML: efficient inference of recombination in whole bacterial genomes. PLoS Comput Biol.

[32] Méric G, Yahara K, Mageiros L, Pascoe B, Maiden MC (2014). A reference pan-genome approach to comparative bacterial genomics: identification of novel epidemiological markers in pathogenic *Campylobacter*. PLoS One.

[33] Shimuta K, Watanabe Y, Nakayama S, Morita-Ishihara T, Kuroki T (2015). Emergence and evolution of internationally disseminated cephalosporin-resistant *Neisseria gonorrhoeae* clones from 1995 to 2005 in Japan. BMC Infect Dis.

[34] Nakayama S, Shimuta K, Furubayashi K, Kawahata T, Unemo M (2016). New ceftriaxone- and multidrug-resistant *Neisseria gonorrhoeae* strain with a novel mosaic penA gene isolated in Japan. Antimicrob Agents Chemother.

[35] Tamura K, Stecher G, Peterson D, Filipski A, Kumar S (2013). MEGA6: molecular evolutionary genetics analysis version 6.0. Mol Biol Evol.

[36] Harrison OB, Clemence M, Dillard JP, Tang CM, Trees D (2016). Genomic analyses of *Neisseria gonorrhoeae* reveal an association of the gonococcal genetic island with antimicrobial resistance. J Infect.

[37] Katoh K, Standley DM (2013). MAFFT multiple sequence alignment software version 7: improvements in performance and usability. Mol Biol Evol.

[38] Benjamini Y YH (1995). Controlling the false discovery rate: a practical and powerful approach to multiple testing. J R Stat Soc Series B.

[39] Demczuk W, Martin I, Peterson S, Bharat A, van Domselaar G (2016). Genomic epidemiology and molecular resistance mechanisms of azithromycin-resistant *Neisseria gonorrhoeae* in Canada from 1997 to 2014. J Clin Microbiol.

[40] Jacobsson S, Golparian D, Cole M, Spiteri G, Martin I (2016). WGS analysis and molecular resistance mechanisms of azithromycin-resistant (MIC >2 mg/L) *Neisseria gonorrhoeae* isolates in Europe from 2009 to 2014. J Antimicrob Chemother.

[41] Hunt M, Mather AE, Sánchez-Busó L, Page AJ, Parkhill J (2017). ARIBA: rapid antimicrobial resistance genotyping directly from sequencing reads. Microb Genom.

[42] Jolley KA, Maiden MC (2010). BIGSdb: scalable analysis of bacterial genome variation at the population level. BMC Bioinformatics.

[43] Hadfield J, Croucher NJ, Goater RJ, Abudahab K, Aanensen DM (2018). Phandango: an interactive viewer for bacterial population genomics. Bioinformatics.

[44] Croucher NJ, Page AJ, Connor TR, Delaney AJ, Keane JA (2015). Rapid phylogenetic analysis of large samples of recombinant bacterial whole genome sequences using Gubbins. Nucleic Acids Res.

[45] Rambaut A, Lam TT, Carvalho LM, Pybus OG (2016). Exploring the temporal structure of heterochronous sequences using TempEst (formerly Path-O-Gen). Virus Evol.

[46] Didelot X, Croucher NJ, Bentley SD, Harris SR, Wilson DJ (2018). Bayesian inference of ancestral dates on bacterial phylogenetic trees. bioRxiv.

[47] Suchard MA, Lemey P, Baele G, Ayres DL, Drummond AJ (2018). Bayesian phylogenetic and phylodynamic data integration using BEAST 1.10. Virus Evol.

[48] Baele G, Lemey P, Suchard MA (2016). Genealogical working distributions for bayesian model testing with phylogenetic uncertainty. Syst Biol.

[49] Rambaut A, Drummond AJ, Xie D, Baele G, Suchard MA Posterior summarisation in Bayesian phylogenetics using Tracer 1.7. Syst Biol.

[50] Vos M, Didelot X (2009). A comparison of homologous recombination rates in bacteria and archaea. ISME J.

[51] Ameyama S, Onodera S, Takahata M, Minami S, Maki N (2002). Mosaic-like structure of penicillin-binding protein 2 gene (penA) in clinical isolates of *Neisseria gonorrhoeae* with reduced susceptibility to cefixime. Antimicrob Agents Chemother.

[52] Unemo M, del Rio C, Shafer WM (2016). Antimicrobial resistance expressed by *Neisseria gonorrhoeae*: a major global public health problem in the 21st century. Microbiol Spectr.

[53] Vincent LR, Kerr SR, Tan Y, Tomberg J, Raterman EL (2018). *In vivo*-selected compensatory mutations restore the fitness cost of mosaic *penA* alleles that confer ceftriaxone resistance in *Neisseria gonorrhoeae*. MBio.

[54] Whittles LK, White PJ, Didelot X (2017). Estimating the fitness cost and benefit of cefixime resistance in *Neisseria gonorrhoeae* to inform prescription policy: a modelling study. PLoS Med.

[55] Ledda A, Price JR, Cole K, Llewelyn MJ, Kearns AM (2017). Re-emergence of methicillin susceptibility in a resistant lineage of *Staphylococcus aureus*. J Antimicrob Chemother.

[56] Chen SC, Yin YP, Dai XQ, Unemo M, Chen XS (2016). First nationwide study regarding ceftriaxone resistance and molecular epidemiology of *Neisseria gonorrhoeae* in China. J Antimicrob Chemother.

[57] Chen Y, Gong Y, Yang T, Song X, Li J (2016). Antimicrobial resistance in *Neisseria gonorrhoeae* in China: a meta-analysis. BMC Infect Dis.

[58] Kunz AN, Begum AA, Wu H, D'Ambrozio JA, Robinson JM (2012). Impact of fluoroquinolone resistance mutations on gonococcal fitness and in vivo selection for compensatory mutations. J Infect Dis.

[59] Vereshchagin VA, Ilina EN, Malakhova MV, Zubkov MM, Sidorenko SV (2004). Fluoroquinolone-resistant *Neisseria gonorrhoeae* isolates from Russia: molecular mechanisms implicated. J Antimicrob Chemother.

[60] Kulkarni S, Bala M, Sane S, Pandey S, Bhattacharya J (2012). Mutations in the gyrA and parC genes of quinolone-resistant *Neisseria gonorrhoeae* isolates in India. Int J Antimicrob Agents.

[61] Turner KM, Christensen H, Adams EJ, McAdams D, Fifer H (2017). Analysis of the potential for point-of-care test to enable individualised treatment of infections caused by antimicrobial-resistant and susceptible strains of *Neisseria gonorrhoeae*: a modelling study. BMJ Open.

[62] Sadiq ST, Mazzaferri F, Unemo M (2017). Rapid accurate point-of-care tests combining diagnostics and antimicrobial resistance prediction for *Neisseria gonorrhoeae* and *Mycoplasma genitalium*. Sex Transm Infect.

[63] Pond MJ, Hall CL, Miari VF, Cole M, Laing KG (2016). Accurate detection of *Neisseria gonorrhoeae* ciprofloxacin susceptibility directly from genital and extragenital clinical samples: towards genotype-guided antimicrobial therapy. J Antimicrob Chemother.

[64] Unemo M, Nicholas RA (2012). Emergence of multidrug-resistant, extensively drug-resistant and untreatable gonorrhea. Future Microbiol.

