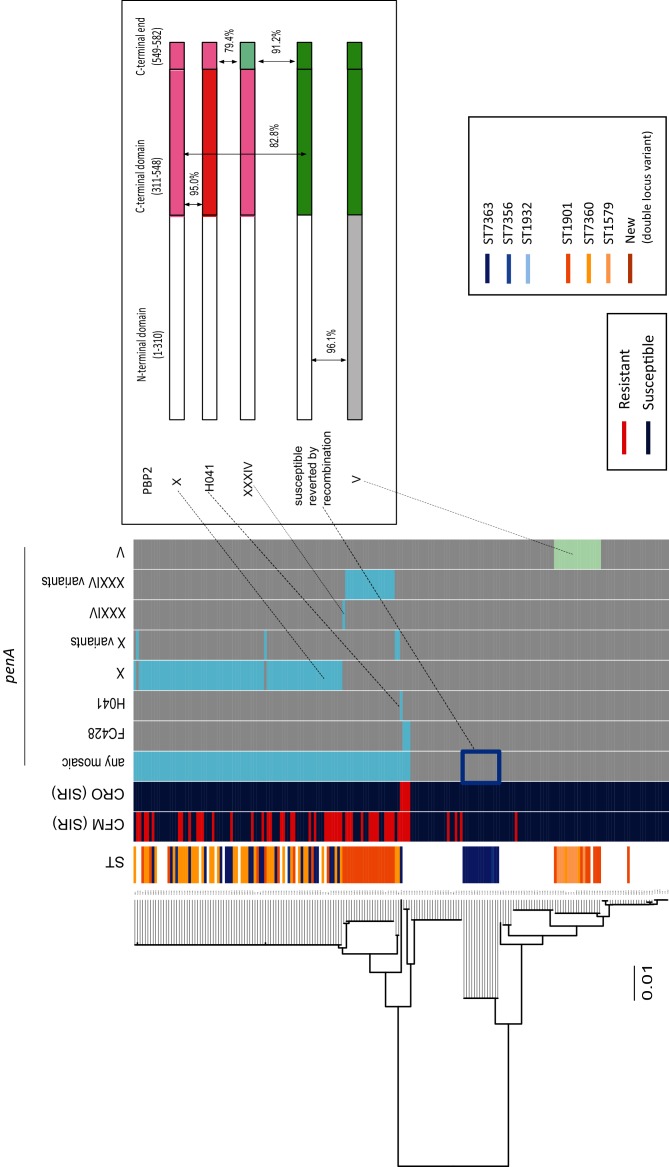# Corrigendum: Genomic surveillance of *Neisseria gonorrhoeae* to investigate the distribution and evolution of antimicrobial-resistance determinants and lineages

**DOI:** 10.1099/mgen.0.000215

**Published:** 2018-09-28

**Authors:** Koji Yahara, Shu-ichi Nakayama, Ken Shimuta, Ken-ichi Lee, Masatomo Morita, Takuya Kawahata, Toshiro Kuroki, Yuko Watanabe, Hitomi Ohya, Mitsuru Yasuda, Takashi Deguchi, Xavier Didelot, Makoto Ohnishi

**Affiliations:** ^1^​ Antimicrobial Resistance Research Center, National Institute of Infectious Diseases, Tokyo, Japan; ^2^​ Department of Bacteriology I, National Institute of Infectious Diseases, Tokyo, Japan; ^3^​ Virology Section, Division of Microbiology, Osaka Institute of Public Health, Osaka, Japan; ^4^​ Department of Microbiology, Kanagawa Prefectural Institute of Public Health, Kanagawa, Japan; ^5^​ Department of Urology, Graduate School of Medicine, Gifu University, Gifu, Japan; ^6^​ Department of Infectious Disease Epidemiology, Imperial College, London, UK; ^†^​ Present address: Faculty of Veterinary Medicine, Okayama University of Science, 1-3, Ikoinooka, Imabari, Ehime 794-8555, Japan.

There was an error in Fig. 2 in the published article.

The positions of the N-terminal domain and C-terminal domain have been corrected:

1. (1–239) corrected to (1–310)
2. (240–548) -> (311–548)
3. Accordingly,
  97.1% -> 96.1%
  96.1% -> 95.0%
  79% -> 82.8%

The corrected Figure is shown below.

The author apologizes for any inconvenience caused.

**Figure:**