# Supplementary material for: Genomic surveillance of Neisseria gonorrhoeae to investigate the distribution and evolution of antimicrobial-resistance determinants and lineages
Source: Microb Genom. 2018 Jul 31;4(8):e000205. doi: 10.1099/mgen.0.000205 (PMC6159555; doi:10.1099/mgen.0.000205)
Supplement: Supplementary File 1 [file mgen-5-205-s001.pdf]

Supplementary Figures

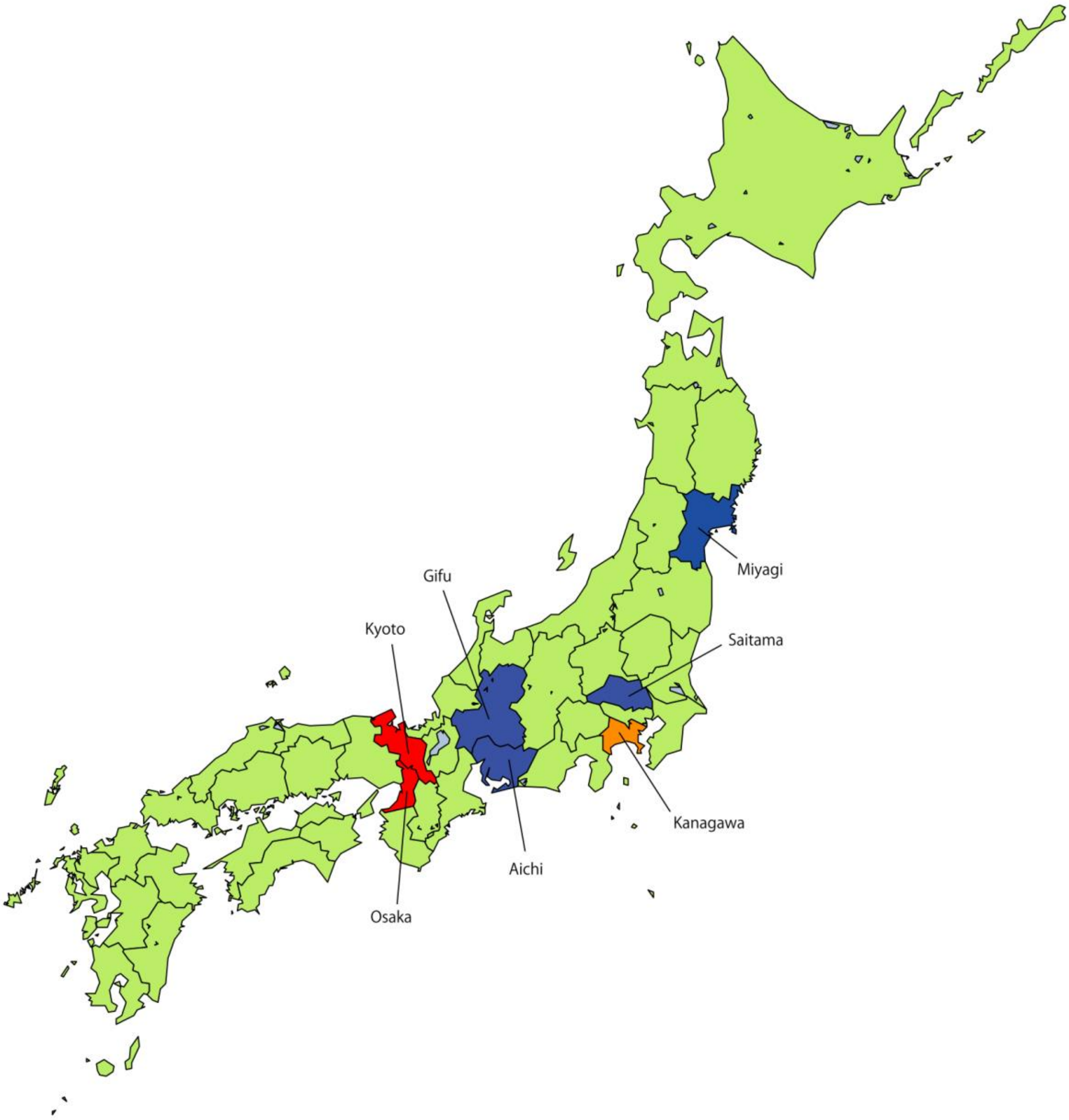

Figure S1. Japanese map. Kyoto and Osaka (red) are the target of the surveillance where the 204 strains were isolated in 2015. Miyagi, Saitama, Aichi, and Gifu (blue) are regions where the additional 40 strains of ST7363 were isolated in 2015. Kanagawa (orange) is a region where the 21 historical strains were isolated from 1996 to 2000.

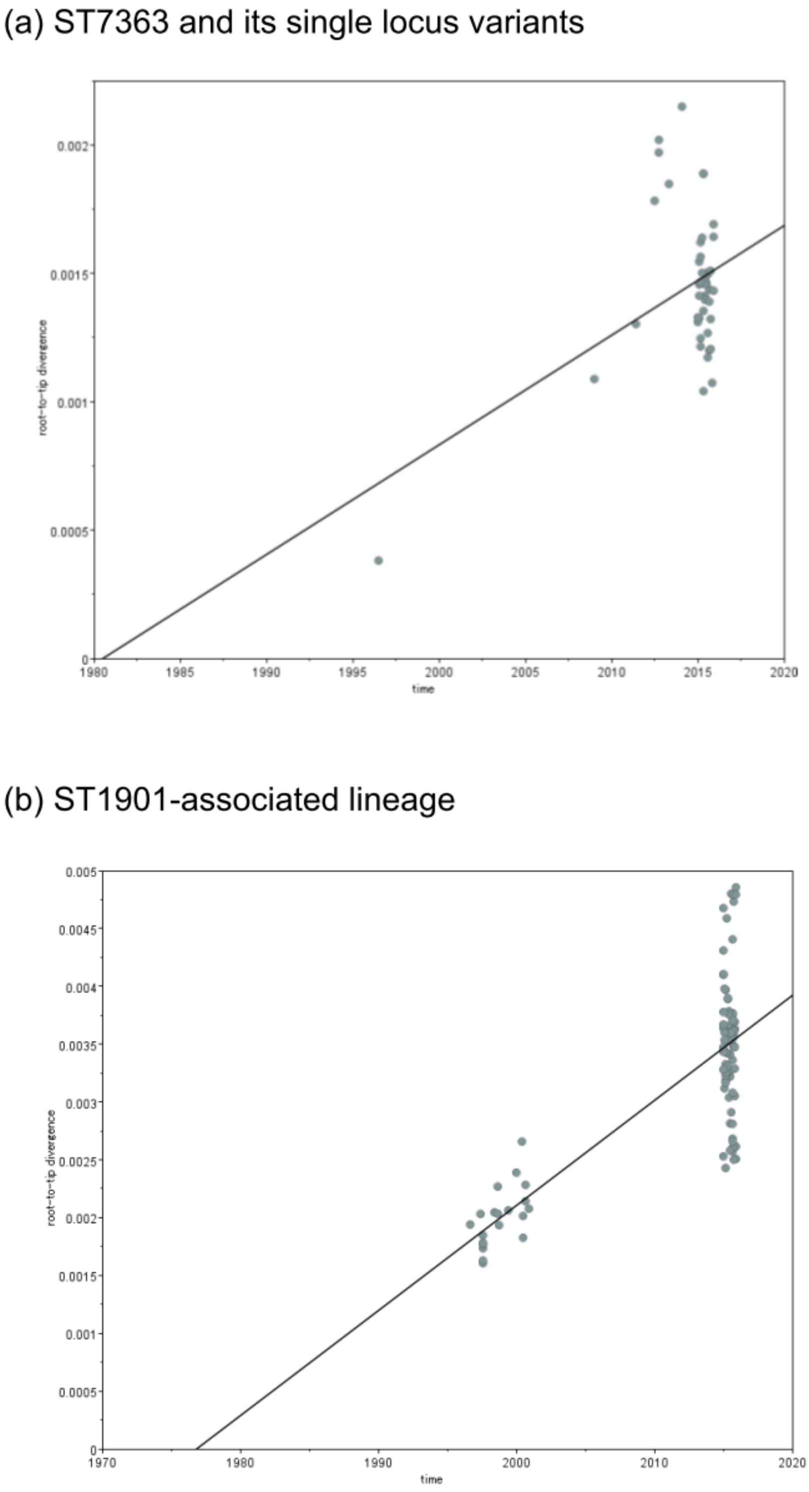

Figure S2. Regressions of sampling time versus root-to-tip genetic distance. (a) ST7363 and its single locus variants. (a) ST7363 and its single locus variants. (b) ST1901-associated lineage.

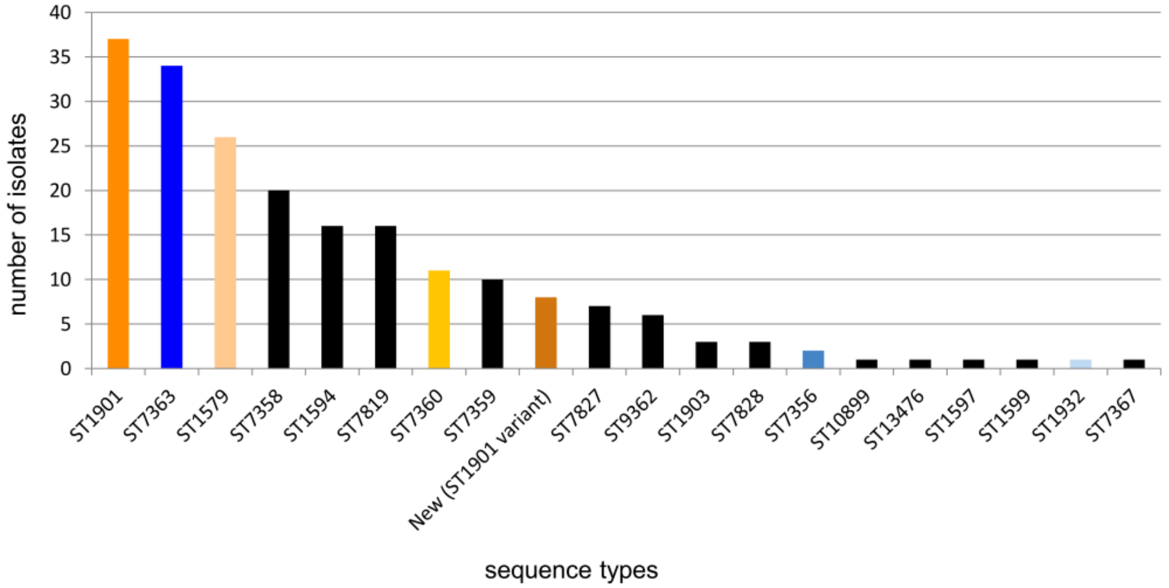

Figure S3. Distribution of sequence types of the 204 strains isolated in Kyoto and Osaka in 2015. The colors except for the black correspond to those in Figure 1, 2, and 3 to highlight specific sequence types.

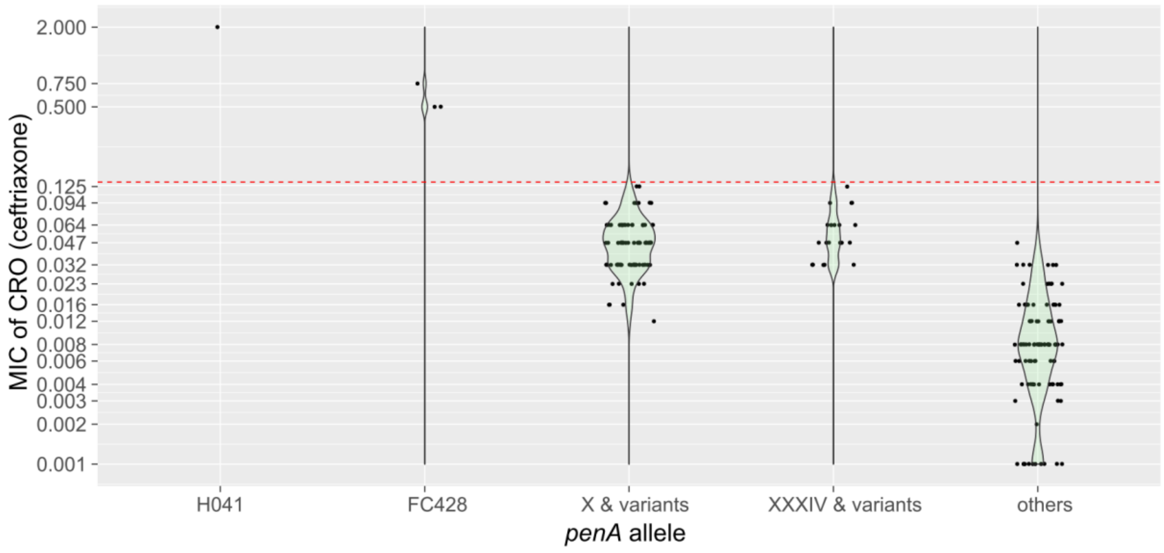

Figure S4. Relationship between *penA* alleles and MIC of ceftriaxone. The y-axis indicates MIC values measured by Etest. Each dot corresponds to each strain, and horizontal width of the green corresponds to the number of strains showing the MIC value. The red dashed line indicates the MIC breakpoint > 0.125 to define resistance phenotype according to EUCAST.

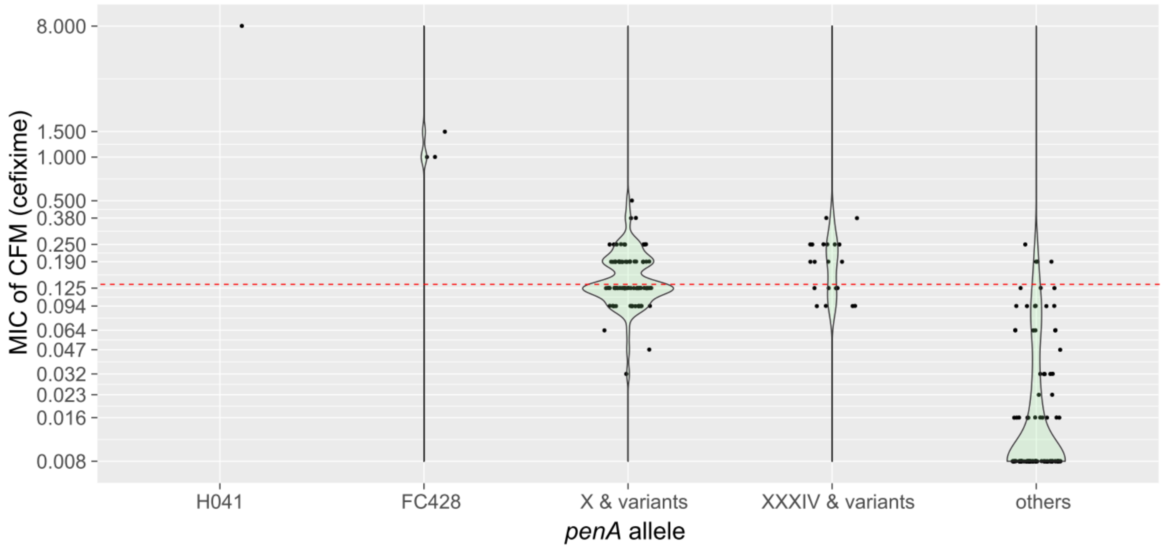

Figure S5. Relationship between *penA* alleles and MIC of cefixime. The y-axis indicates MIC values measured by Etest. Each dot corresponds to each strain, and horizontal width of the green corresponds to the number of strains showing the MIC value. The red dashed line indicates the MIC breakpoint > 0.125 to define resistance phenotype according to EUCAST.

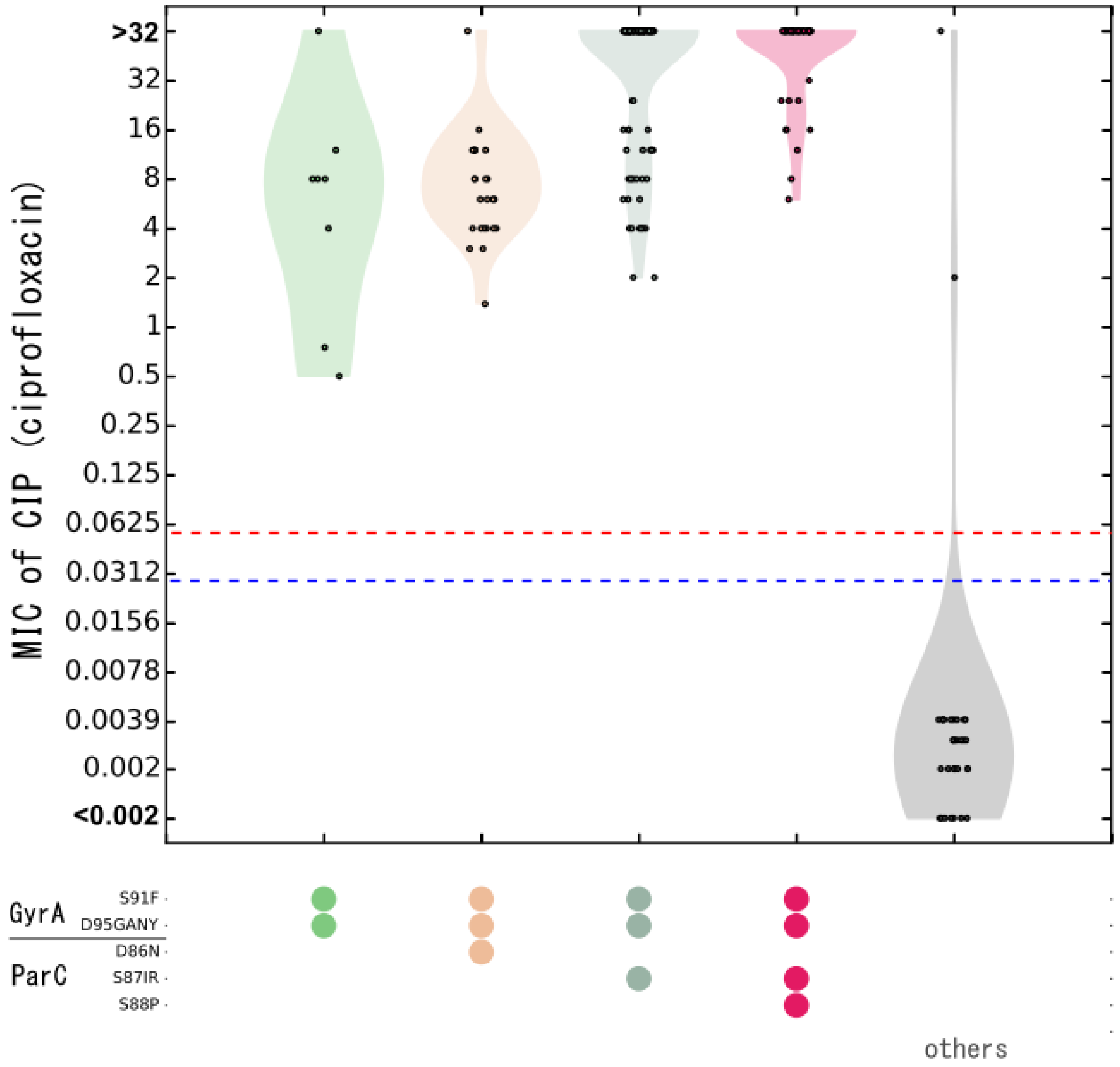

Figure S6. Relationship between substitutions in GyrA and ParC and MIC of ciprofloxacin. The y-axis indicates MIC values measured by Etest. Each dot corresponds to each strain, and horizontal width of the green corresponds to the number of strains showing the MIC value. The red and blue dashed lines indicate the MIC breakpoints > 0.06 and ≤ 0.03 to define resistance and susceptible phenotypes according to EUCAST.

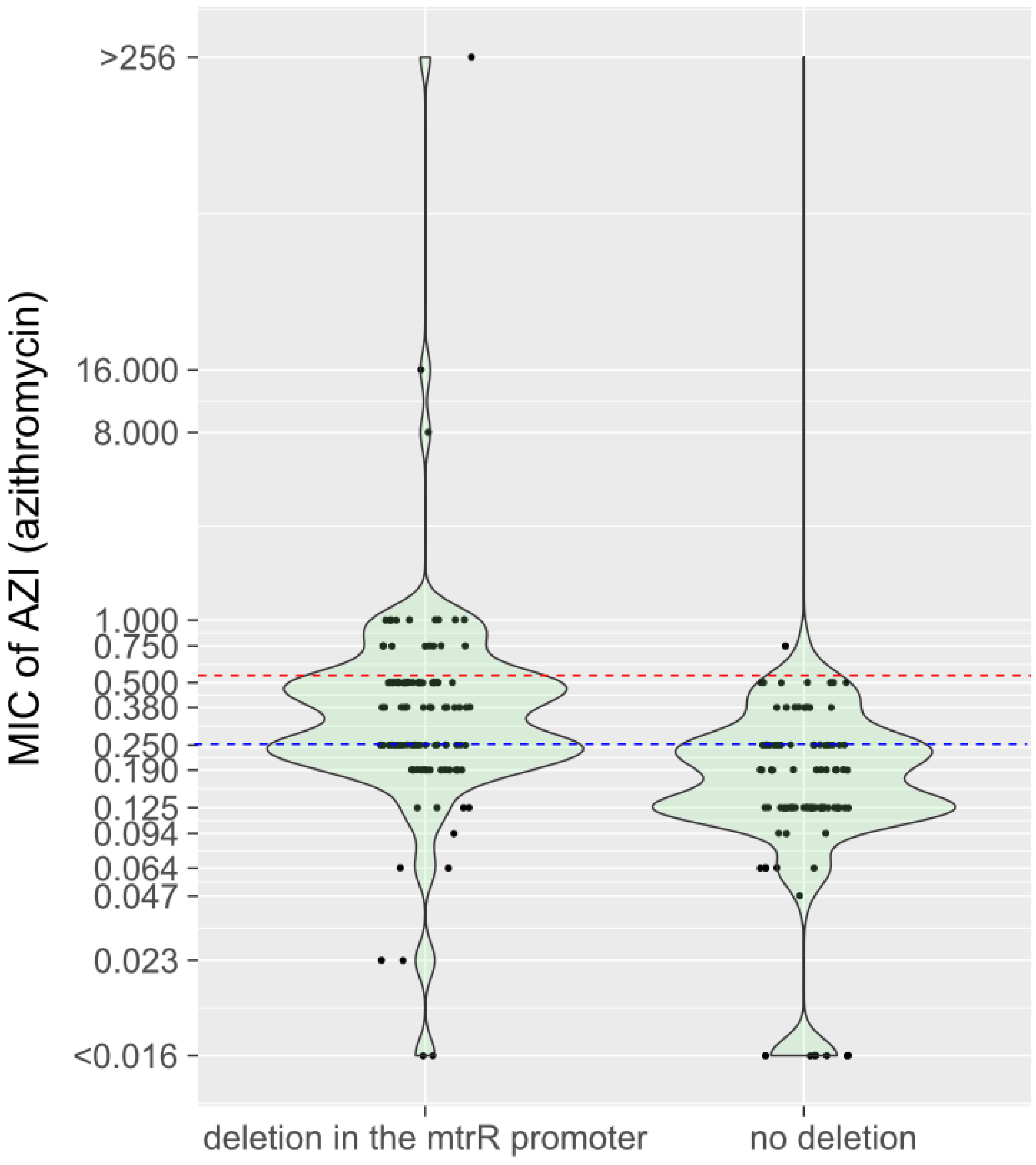

Figure S7. Relationship between the adenosine deletion in the promoter region of *mtrR* and MIC of azithromycin. The y-axis indicates MIC values measured by Etest. Each dot corresponds to each strain, and horizontal width of the green area corresponds to the number of strains showing the MIC value. The red and blue dashed lines indicate the MIC breakpoints > 0.5 and ≤ 0.25 to define resistance and susceptible phenotypes according to EUCAST.
